# Affinity purification mass spectrometry characterisation of the interactome of receptor tyrosine kinase proline-rich motifs in cancer

**DOI:** 10.1016/j.heliyon.2024.e35480

**Published:** 2024-07-31

**Authors:** Christopher M. Jones, Arndt Rohwedder, Kin Man Suen, Safoura Zahed Mohajerani, Antonio N. Calabrese, Sabine Knipp, Mark T. Bedford, John E. Ladbury

**Affiliations:** a Department of Oncology, University of Cambridge, Cambridge, CB2 0XZ, UK; b Faculty of Biological Sciences, University of Leeds, Leeds, LJ2 9JT, UK; c Centre for Medical Research (ZMF), Johannes Kepler University, 4020 Linz, Austria; d Department of Epigenetics & Molecular Carcinogenesis, University of Texas MD Anderson Cancer Centre, Houston, TX. TX 77230, USA

**Keywords:** Receptor tyrosine kinase (RTK), SRC homology domain 3 (SH3), SRC homology domain 2 (SH2), Proline-rich motif (PRM), Cancer

## Abstract

Receptor tyrosine kinase (RTK) overexpression is linked to the development and progression of multiple cancers. RTKs are classically considered to initiate cytoplasmic signalling pathways via ligand-induced tyrosine phosphorylation, however recent evidence points to a second tier of signalling contingent on interactions mediated by the proline-rich motif (PRM) regions of non-activated RTKs. The presence of PRMs on the C-termini of >40 % of all RTKs and the abundance of PRM-binding proteins encoded by the human genome suggests that there is likely to be a large number of previously unexplored interactions which add to the RTK intracellular interactome. Here, we explore the RTK PRM interactome and its potential significance using affinity purification mass spectrometry and in silico enrichment analyses. Peptides comprising PRM-containing C-terminal tail regions of EGFR, FGFR2 and HER2 were used as bait to affinity purify bound proteins from different cancer cell line lysates. 490 unique interactors were identified, amongst which proteins with metabolic, homeostatic and migratory functions were overrepresented. This suggests that PRMs from RTKs may sustain a diverse interactome in cancer cells. Since RTK overexpression is common in cancer, RTK PRM-derived signalling may be an important, but as yet underexplored, contributor to negative cancer outcomes including resistance to kinase inhibitors.

## 1 Introduction

Receptor tyrosine kinases (RTKs) are key mediators of intracellular signals controlling cellular growth, proliferation and motility [1]. Stimulation of transmembrane RTKs by a cognate extracellular ligand generally results in homo- or hetero-dimerization and subsequent autophosphorylation of tyrosine (pTyr) residues within cytoplasmic C-terminal RTK tails [1]. These form docking sites for the binding of Src homology 2 (SH2), phosphotyrosine binding (PTB) and other-related domains. The development and progression of a wide range of malignancies are linked to signalling derived from RTKs [2]. To date, autophosphorylation of RTKs has been considered the predominant mediator of their oncogenic potential [2]. Despite this, therapeutic strategies to prevent RTK autophosphorylation, such as through the use of directed small molecule tyrosine kinase inhibitors, are not universally successful [3]. Further, in many cases, oncogenesis is linked to RTK protein overexpression rather than directly to increased RTK activation [2].

**Fig. 1:**
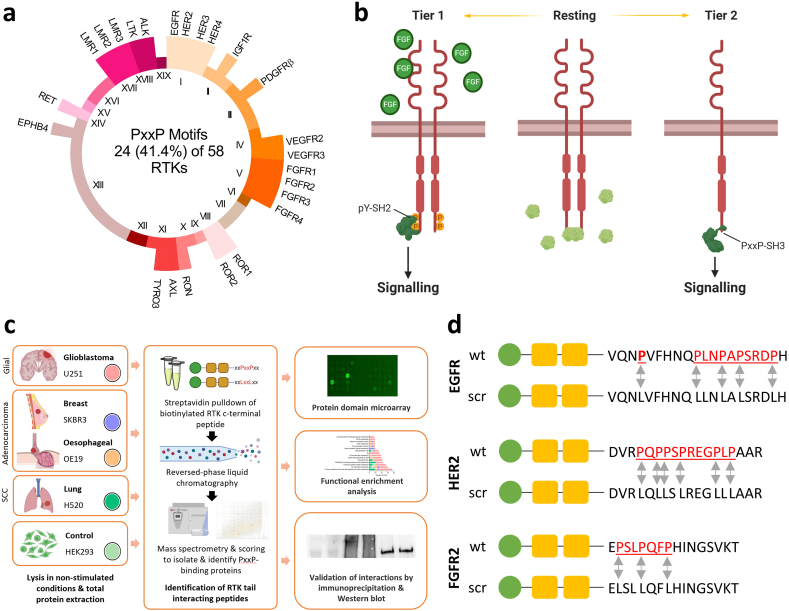
**Streptavidin affinity pulldown identifies binding partners for the proline-rich C-terminal tail sequences of the receptor tyrosine kinases (RTKs) epidermal growth factor receptor (EGFR), fibroblast growth factor receptor 2 (FGFR2) and erb-B2 (ERBB2/HER2). (a)** A schematic overview of the prevalence of proline-rich motifs (PRMs) within each of the nineteen RTK groups encoded by the human genome. All 58 RTKs contribute to the circle equally, with only the 24 that incorporated a PRM labelled. Tail sequences are summarised in Supplementary Table 1 (b) A schematic of the proposed tiers of RTK-derived signalling is shown using FGFR2. The *resting* state represents conditions of low ligand availability but a relative excess of the adaptor protein growth factor receptor bound protein 2 (GRB2), which binds via SH3 domains to proline-rich sequences on the cytoplasmic tail of the RTK to form a stable heterotetramer. *Tier 1* refers to a canonical signalling mechanism through which ligand stimulation (or an activating mutation (not shown)) results in autophosphorylation of cytoplasmic C-terminal tail tyrosine residues, to which effector proteins bind via Src Homology 2 (SH2) or phosphotyrosine binding (PTB) domains. *Tier 2* represents conditions of low ligand availability but a relative excess of RTK compared with GRB2, such as may occur following RTK protein overexpression in instances of gene amplification. In these conditions, binding of effector proteins to RTK PRMs via SH3 and related domains results in downstream signalling. **(c)** A summary of the experimental approach used to identify binding partners of PRM-containing tail regions of EGFR, FGFR2 and HER2 in cells representing glioblastoma (U251), lung squamous cell carcinoma (H520), oesophageal adenocarcinoma (OE19), breast adenocarcinoma (SK-BR-3) or a control cell line (HEK293T) that stably overexpresses FGFR2. Lysates from each cell line were individually incubated with bait peptides representing PRM-containing wild-type RTK C-terminal tail sequences or scrambled control sequences in which proline residues were replaced with leucine and which were devoid of tyrosine residues. Mass-spectrometry was used to identify bound peptides, which were identified using MaxQuant and compared across bait and control tail sequences using SAINTexpress and Perseus. Functional enrichment of identified interactors was used to characterise their structure and function. A subset of interactors were evaluated through live cell imaging to confirm their proximity and by gene knockdown analysis in order to assay their function in conditions of low RTK phosphorylation. **(d)** Bait peptide sequences used for the streptavidin pulldown. Tail sequences of EGFR (residues 1105–1124), HER2 (residues 1141–1161) and FGFR2 (residues 806–821) were covalently bound to biotin via two polyethylene glycoPEG) spacers. Proline residues and PRMs are underlined and highlighted in red. Arrows are used to signify points at which proline residues in the wild-type (wt) bait polypeptide were replaced by leucine residues within scrambled (scr) control bait polypeptide for each of the three RTK sequences. (For interpretation of the references to color in this figure legend, the reader is referred to the Web version of this article.)

Binding of proline-rich sequences by Src homology 3 (SH3) domains is critical to the assembly of a number of signalling complexes [[4], [5], [6], [7], [8], [9], [10], [11], [12], [13]]. A comprehensive study mapping the physical and functional interactome of human RTKs identified SH3 domain-containing proteins as those most commonly bound; albeit without providing evidence for their binding sites [14]. Of the 58 RTKs encoded by the human genome, 24 feature a canonical proline-rich motif (PRM) capable of recognising SH3 domains within their cytoplasmic C-terminal tail sequence (Fig. 1a, Supp. Table 1) [15]. Combined with the identification of in excess of 300 sequences for SH3 domains across over 200 different proteins expressed in humans [16] there exists the potential for a multitude of previously unstudied interactions.

Recent evidence to indicate that the recruitment of signalling proteins to these sites, in the absence of RTK upregulation, associates with signalling activity and pathological outcomes, but the extent to which this defines cancer outcomes is unclear [[17], [18], [19], [20]]. A notable exception is the known interaction of the C-terminal SH3 domain of the adaptor protein growth factor receptor bound protein 2 (GRB2) and the phospholipase Cγ1 (PLCγ1) with a PRM within the C-terminal tail of fibroblast growth factor receptor 2 (FGFR2) [17,18]. In the absence of RTK activity, intracellular, concentration-dependent competition between the SH3 domains of GRB2 and PLCγ1 for the receptor PRM regulates activity of the phospholipase and AKT-mediated cell proliferation and motility [19,20]. There is, in addition, evidence for SH3 domain-mediated binding of the proto-oncogene protein tyrosine kinase, FYN, to a cytoplasmic C-terminal tail PRM within the ERBB2 (HER2) receptor tyrosine kinase [21]. Other domains are also recognised to bind PRMs but have not yet been explored in the context of RTK PRM sequences. These include WW domains, Ena/Vasp homology domain 1 (EVH1) domains, glycine-tyrosine-phenylalanine (GYF) domains, ubiquitin E2 variant (UEV) domains and single-domain profilin proteins [22].

Together, these findings suggest the presence of a second tier of RTK-derived signalling that is not contingent on ‘on/off’ ligand-induced pTyr-mediated signalling, but on interactions that occur in the absence of ligand stimulation by C-terminal PRMs (Fig. 1b). Crucially, this signalling (henceforth termed Tier 2) is dependent on the relative intracellular concentration of cognate effector proteins. This means that conditions that drive fluctuations in concentration of these proteins (e.g., environmental stress) will permit proteins to prevail in interactions with specific RTKs and initiate different patterns of signalling. Within the context of cancer, most RTK-related research has been focused on activating mutations that hyperstimulate pTyr-mediated Tier 1 signalling [2]. There is, however, accumulating evidence that a number of cancers are characterised, and their outcomes, at least in part, dictated by frequent protein overexpression of RTKs [2]. This occurs through diverse processes including genomic amplification, loss of negative regulation and the increased transcription and translation of RTK-encoding genes [2,3]. The result is a significant increase within a cell in the local concentration of overexpressed RTKs, many of which harbour PRMs capable of mediating non-canonical Tier 2 signalling.

Despite the potential importance of this to cancer outcomes, the pathways through which RTK PRMs mediate signalling are not known. Given this, we sought to uncover and characterise the PRM interactome of the RTKs epidermal growth factor receptor (EGFR), FGFR2 and ERBB2/HER2 in cell lines resembling oesophageal adenocarcinoma (OAC), breast adenocarcinoma (BrAC), glioblastoma (GBM) and lung squamous cell carcinoma (LSCC). These are malignancies in which amplification or overexpression of the three chosen RTKs is frequently identified and has been shown to be associated with survival outcomes [2,[23], [24], [25], [26], [27], [28], [29], [30], [31], [32]]. In studying these, we provide evidence for a diverse RTK PRM interactome that is enriched for metabolic, homeostatic and pro-migratory signalling pathways. We also provide further evidence for the importance to cancer outcomes of SH3-mediated interactions with the PRM of RTKs in the absence of pTyr upregulation.

## 2 Results

### 2.1 The RTK PRM-containing tail region interactome across RTKs and cancer cell lines

In order to uncover the interactomes for specific RTK PRMs, a PRM-incorporating, tyrosine depleted, C-terminal tail regions from each of the RTKs; EGFR, FGFR2 and ERBB2/HER2 was used as bait to affinity purify bound proteins in cell lines resembling OAC, BrAC, GBM and LSCC as well as a non-cancerous cell line (HEK293T); as summarised in Fig. 1c. Captured proteins were compared to those bound to similar bait peptides in which PRMs were replaced by leucine residues (Fig. 1d). The inclusion of leucine residues precludes the PRM from adopting the canonical PPII helical structure required for ligand recognition. PRM-interacting proteins were subsequently identified by high-resolution mass spectrometry.

**Fig. 2:**
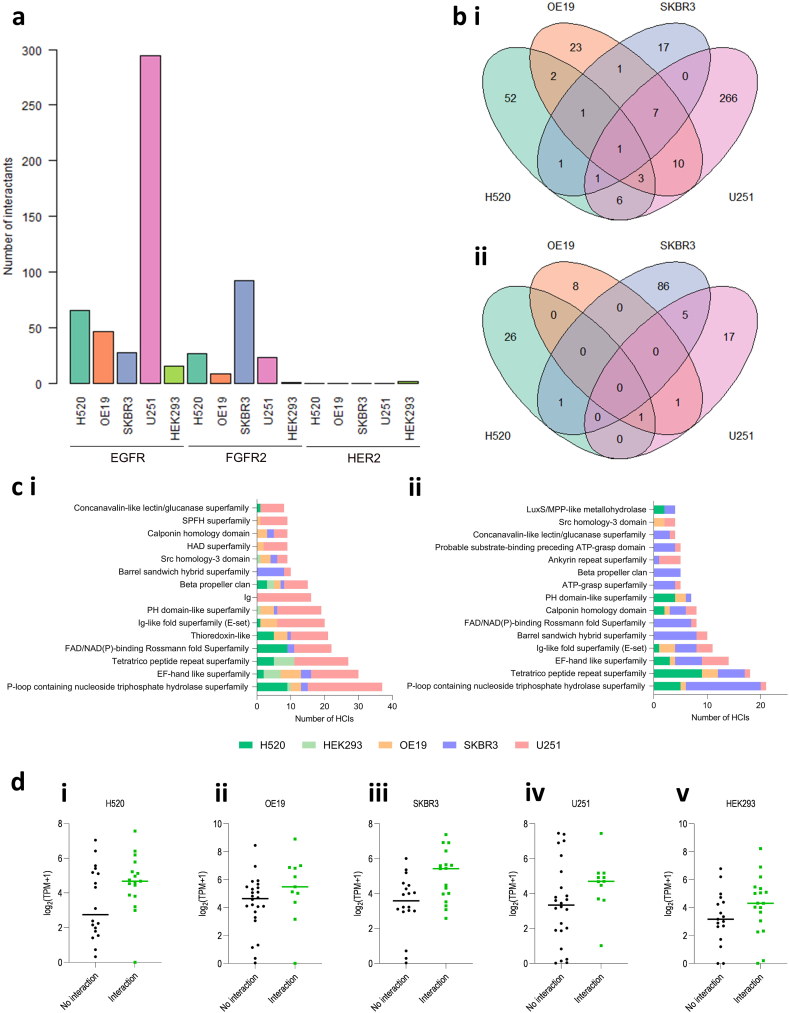
**A summary of the number and structural composition of interactors for proline-rich C-terminal sequences of the receptor tyrosine kinases (RTKs) epidermal growth factor receptor (EGFR), fibroblast growth factor receptor 2 (FGFR2) and erb-B2 (ERBB2/HER2). (a)** The total number of interactors for each streptavidin pulldown experiment using a proline-rich C-terminal RTK tail sequence is shown for each of the studied cell lines: lung squamous cell carcinoma (H520), oesophageal adenocarcinoma (OE19), breast adenocarcinoma (SKBR3), glioblastoma (U251) and human embryonic kidney (HEK293T). **(b)** Venn diagrams illustrating the number of interacting proteins for the **(i)** EGFR and **(ii)** FGFR2 proline-rich C-terminal tail sequences that were shared across each of the four studied cell lines. There were no shared interactors for the HER2 C-terminal tail sequence. **(c)** A summary of the most commonly identified Pfam protein clans for the **(i)** EGFR and **(ii)** FGFR2 proline-rich C-terminal tail sequences and their relationship to the studied cell lines. **(d)** mRNA expression data were extracted for each studied cell line from the Cancer Cell Line Encyclopedia for the cohort of SH3 domain-containing interactors shown in Table 2. Expression data, shown as a pseudo-count of log2(transcripts per million (TPM)+1), are categorised by the interaction status of the protein to which they correspond. Genes corresponding to proteins not identified as an interactor for any of the studied RTKs in each specific cell line are categorised as ‘No interaction’ and genes corresponding to proteins identified as an interactor for at least one of the studied RTKs in each cell line are listed as ‘Interaction’. Data are shown for each of **(i)** H520, **(ii)** OE19, **(iii)** SKBR3, **(iv)** U251 and **(v)** HEK293T cell lines.

Using the probabilistic SAINTexpress scoring algorithm, a total of 490 unique proteins were identified as interactors for at least one RTK PRM-containing tail region in at least one of the studied cell lines (Fig. 2a, Supp. Tables 2–17) (see Methods). Across all five studied cell lines, the largest number of interactors was seen with the EGFR PRM-tail region (n = 454). In contrast, 155 interactors were identified for the FGFR2 PRM tail region and only two for the HER2 PRM tail region. For the EGFR PRM-tail region, the greatest number of interactors were seen for U251 cells (n = 294), with 67 identified for H520 cells, 48 for OE19 cells and 29 for SKBR3 cells. For the FGFR2 PRM-tail region, 92 interactors were identified for SKBR3 cells, 28 for H520 cells, 24 for U251 cells and ten for OE19 cells. The identified number of interactors was lowest in HEK293T cells for both the EGFR (n = 16) and the FGFR2 (n = 1) PRM-tail regions. This discrepancy in the number of interactors identified for each cell lysate is consistent with the different expression profiles of PRM-binding proteins which presents a unique repertoire of signalling proteins at distinct concentrations available that each PRM can accommodate.

In total, sixty proteins bound both the EGFR and the FGFR2 proline-rich motifs: 28 (46.7 %) within the same cell lines (Supp. Table 2). One interactor (triosephosphate isomerase, TPIS) was identified as bound to EGFR in each of the cancerous cell lines, whereas there was no consistently identified interactor for FGFR2 or HER2 (Fig. 2b). Thirty-three proteins were interactors for the EGFR PRM in more than one cell line, whereas three proteins were interactors for the FGFR2 and none for HER2 PRMs in more than one cell line. The two interacting proteins for HER2 (Treacle protein, TCOF; Dedicator of cytokinesis protein 7, DOCK7) - both of which were identified in HEK293T cells - were not identified as potential interactors for either EGFR or FGFR2. Across the studied RTKs and cell lines, the most frequently identified protein was the cytoskeletal protein Spectrin alpha chain, non-erythrocytic 1 (SPTAN1), which bound both the EGFR and FGFR2 PRM-tail regions in OE19 and U251 cells, in addition to the EGFR PRM-tail region in SKBR3 cells.

We characterised the protein interactors of each studied RTK PRM by classifying them by Pfam clan (Fig. 2c–Supp. Table 18). The P-loop-containing nucleoside triphosphate hydrolase superfamily, tetratrico peptide repeat superfamily and EF-hand like superfamily were the most frequently identified amongst the EGFR and FGFR2 interactors. The tetratrico peptide repeat superfamily was also represented by one of the two HER2 interactors.

We also sought to evaluate whether the expression level of identified interactors influences the probability of their identification as a cell interactor. To do so, mRNA expression of genes encoding the full list of identified RTK PRM interactors (Supp. Table 2) across all RTKs and cell lines was obtained for each cell line from The Cancer Cell Line Encyclopedia (CCLE) [33]. These are correlated against interaction status for each cell line in Fig. 2d, such that counts for genes encoding proteins not identified as an interactor for any of the studied RTKs in each cell line are grouped as ‘No interaction’ and counts for genes encoding proteins identified as an interactor for at least one of the RTKs in each cell line are grouped as ‘Interaction’. A higher median expression score was seen in each cell line for interactors, suggesting that relative protein concentration influences the probability of a PRM-SH3 interaction occurring.

These data reveal that PRMs from a subset of RTKs are able to interact with a large and diverse range of proteins from cancer cell lysates. If this PRM-mediated interactome is replicated in vivo this represents a substantial and previously over-looked signal regulating capability.

### 2.2 Metabolic, homeostatic and migratory processes are overrepresented amongst interactors of RTK PRM regions

To evaluate the functional significance of Tier 2 signalling derived from RTK PRMs we identified overrepresented protein class, biological process and molecular function terms amongst interactors for the three RTK-cell line combinations demonstrating the highest number of interactors: the EGFR C-terminal tail in H520 LSCC cells and U251 GBM cells, and the FGFR2 C-terminal tail in SKBR3 BrAC cells.

**Fig. 3:**
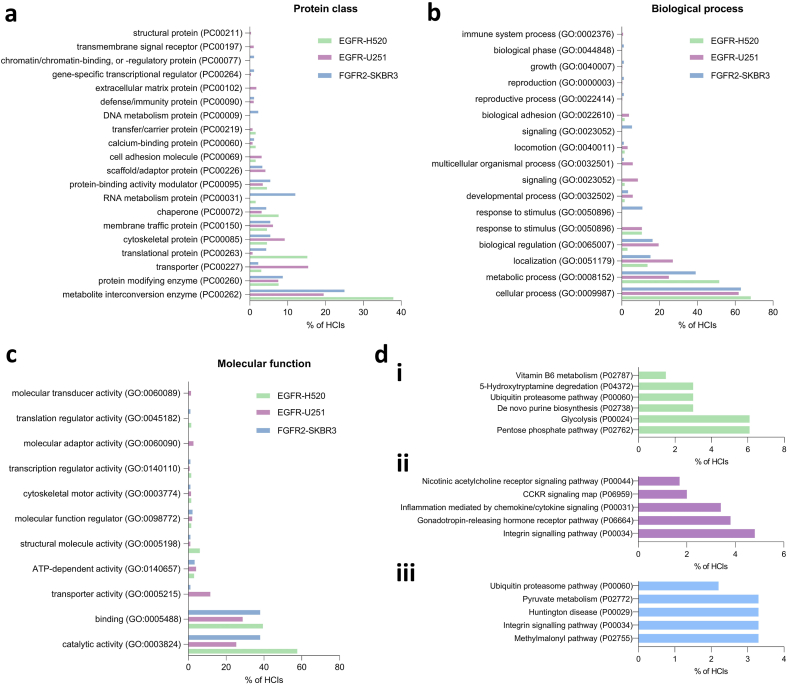
**Functional characterisation of interactors of the epidermal growth factor receptor (EGFR) and fibroblast growth factor receptor 2 (FGFR2) C-terminal tail proline-rich motifs (PRMs) in H520 lung squamous cell carcinoma, U251 glioblastoma and SKBR3 breast adenocarcinoma cells. (a)** Overrepresented protein class terms amongst interactors for EGFR and FGFR2 in H520, U251 and SKBR3 cells. **(b)** Overrepresented biological process terms amongst interactors for EGFR and FGFR2 in H520, U251 and SKBR3 cells. **(c)** Overrepresented molecular function terms amongst interactors for EGFR and FGFR2 in H520, U251 and SKBR3 cells. **(d)** The top 5 overrepresented signalling pathways for interactors of **(i)** the EGFR C-terminal tail PRM in H520 cells, **(ii)** the EGFR C-terminal tail PRM in U251 cells, and **(iii)** the FGFR2 C-terminal tail PRM in SKBR3 cells. In all cases, the proportion of interactors contributing to overrepresentation of each term is shown on the x-axis.

The largest proportion of interactors across each of the studied RTK-cell line combinations were classed as metabolite interconversion or protein modifying enzymes (Fig. 3a). Other commonly identified interactors included translational and cytoskeletal proteins, scaffold/adaptor and chaperone proteins, and cytoskeletal proteins. Accordingly, amongst the interactors, the most overrepresented GO biological processes related to metabolism (‘metabolic process’), homeostasis (‘biological regulation’, ‘response to stimulus’) and cellular movement (‘localisation’, ‘locomotion’ and ‘biological adhesion’); as summarised in Fig. 3b. In keeping with an ability to transduce signalling, ‘catalytic activity’ was the most overrepresented GO molecular function term across each three RTK-cell line combinations (Fig. 3c).

The specific pathways overrepresented by interactors were mostly in keeping with these broad processes (Fig. 3di-iii). This includes metabolic pathways such as those relating to glycolysis, pyruvate metabolism and the Kreb's cycle (methylmalonyl pathway), as well as migration-related pathways such as those relating to integrin signalling. Interestingly, in keeping with overrepresentation of immune system processes amongst interactors of the EGFR PRM in U251 GBM cells, enriched pathways amongst these interactors included ‘inflammation mediated by chemokine/cytokine signalling’ and cholecystokinin receptor ‘CCKR’ signalling (Fig. 3dii).

### 2.3 Analysis of Tier 2 signalling mediator interactions with RTK PRM regions

Since they provide recognition sequences for a range of different protein domains, we analysed interactors for the WW, EVH1, GYF, UEV and profilin domains (Table 1) that are known to interact with PRMs; albeit not in the context of an RTK C-terminal tail. Nineteen EGFR interactors and seven FGFR2 interactors contained an EVH1 domain, as represented by the PH domain-like superfamily. A further four interactors of EGFR featured a profilin domain, as represented by the profilin-like superfamily. There were no recognised WW, GYF or UEV domains amongst the identified interactors.

**Table 1:**
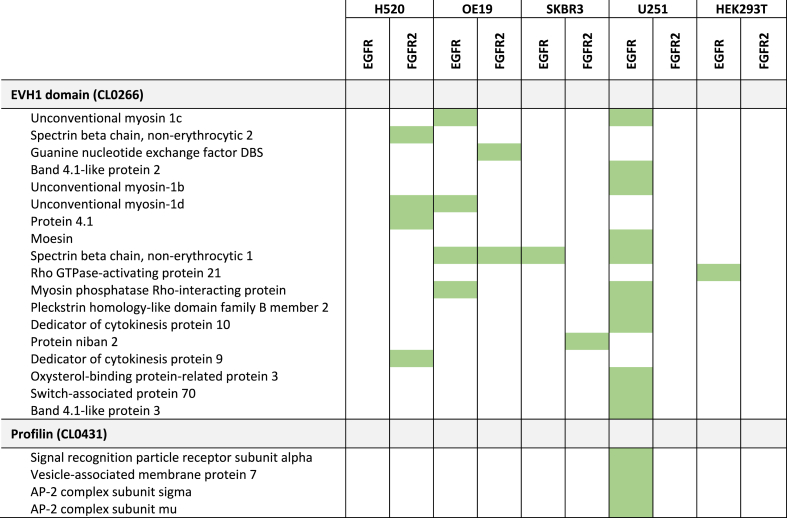
**A summary of identified proline-rich motif (PRM) interactors that incorporate a EVH1 or profilin domain.** The EVH1 and profilin domains have previously been reported to mediate binding to PRMs. Highlighted green cells indicate the presence of a detected interaction between the named protein, listed by the relevant incorporated domain, and the C-terminal tail region of either epidermal growth factor receptor (EGFR) or fibroblast growth factor receptor 2 (FGFR2) in lysates from cells resembling glioblastoma (U251), adenocarcinoma of the lung (H520), oesophagus (OE19) or breast (SKBR3), or from the non-malignant HEK293T cell line. Interactors were identified using SAINTexpress (see Methods).

We also sought to characterise interactions between RTK PRMs and SH3 domain-containing proteins (Table 2). This was of particular interest given previous evidence for a role for SH3-PRM interactions in mediating deleterious cancer outcomes [19,20]. From our data, only six SH3-containing proteins were identified using SAINTexpress as interactors for at least one RTK PRM-containing tail region in at least one of the studied cell lines. This low number of reflects that interactions between PRMs and SH3 domains (K_d_ = 1–100 μM) are at least ten-fold weaker than those of other RTK C-termini interactions (e.g., pTyr sites with SH2 or PTB domains) [34]. The lower affinity of the interactions does not preclude their physiological importance in signalling because, as stated above, the interactions are equilibrium-based and hence dependent on respective concentrations of binding partners.

**Table 2:**
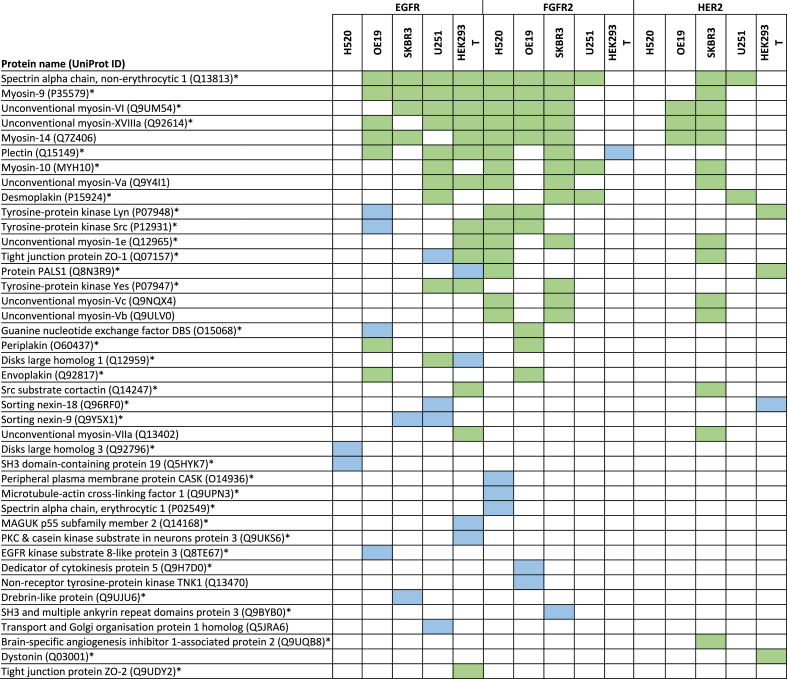
**A summary of SH3 domain-containing interactors (green) and additional low confidence interactors (LCIs; blue) for each of epidermal growth factor receptor (EGFR), fibroblast growth factor receptor 2 (FGFR2) and erb-B2 (ERBB2/HER2) in cell lines representing squamous cell lung cancer (H520), oesophageal adenocarcinoma (OE19), breast adenocarcinoma (SKBR3), glioblastoma (U251) and in a control HEK293T cell line.** Interactors (green) were identified using SAINTexpress and Perseus, with conventional cut off scores applied. Additional LCIs (blue) were identified by SAINTexpress using less stringent cut-off values. Proteins labelled with the GO cellular component identifier ‘plasma membrane’ are highlighted with an asterix.

Importantly, it should also be appreciated that interactions of SH3 domains are typically at least an order of magnitude weaker affinity than interactions usually identified using the AP-MS approach. Consequently, SH3 domain-containing proteins may be underrepresented amongst interactors as determined by the probabilistic SAINTexpress scoring algorithm. Since there is an extensive literature characterising individual interactions of intracellular PRMs and SH3 domains, adopting the stringency level imposed by the SAINTexpress method would deny the existence of these interactions. The data presented here support this, with the Src family tyrosine kinases SRC, LYN and YES proteins not classified as interactors by SAINTexpress despite substantial previous characterisation of their interaction with PRMs [35,36] (Table 2).

Given this, we further scrutinised our data using a two-pronged approach to identify additional SH3 domain-containing protein interactors: (1) using label-free quantitation of on MS-intensity based data (rather than spectral counts as implemented in SAINTexpress) and (2) using less-stringent SAINTexpress cut-off values that were tuned to allow identification of interactors that had been previously experimentally validated (see Methods). Using this approach SRC, LYN and YES were all identified as potential interactors, consistent with previous experimental data. To distinguish identification of interactors using less-stringent SAINTexpress cut-off values from the previously described data, we term these low confidence interactors (LCIs).

We explored this expanded list of interactors to identify SH3-containing proteins and were able to identify a combined total of 41 SH3-containing interactors for at least one of the RTKs in one of the studied cell lines (Table 2). Importantly, of the 122 observed pairwise PRM-SH3 interactions, only 26 (21.3 %) were LCIs. The remaining 96 (78.7 %) were identified using conventional SAINTexpress (n = 5; 4.1 %) or Perseus (n = 81; 66.4 %) scores, with five (4.1 %) interactors identified by both scoring systems and a further five (4.1 %) identified as interactors by Perseus but only as LCIs by SAINTexpress. This points to a potential greater sensitivity for low affinity reactions for Perseus over SAINTexpress [37].

**Fig. 4:**
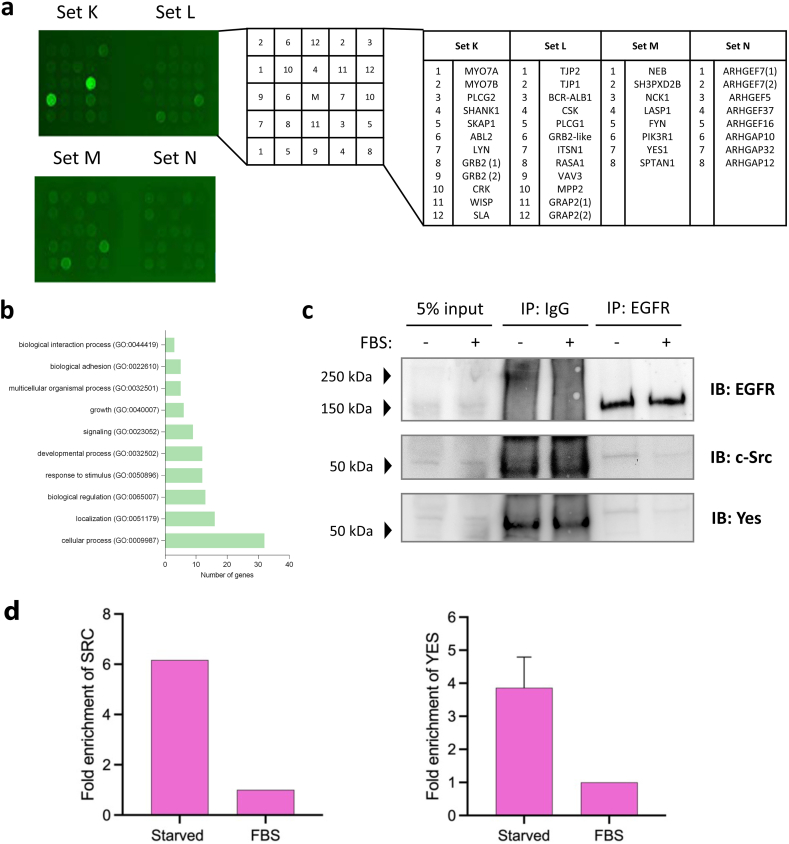
**A functional overview of Src homology 3 (SH3) domain-containing PRM interactors for each of epidermal growth factor receptor (EGFR), fibroblast growth factor receptor 2 (FGFR2) and ERBB2 (HER2). (a)** A peptide microarray demonstrating interactions between the C-terminal tail of HER2 and glutathione S-transferase (GST) fusion SH3 domain-containing proteins spotted on to a positional grid. The correlation between each protein and its grid position is shown alongside. Bound peptides are those with two fluorescent spots present. The control peptide microarray is shown in Supp. Fig. 1. (b) Overrepresented GO biological process terms amongst interacting SH3 domain-containing peptides. **(c)** Immunoprecipitation demonstrating that EGFR forms a protein complex with c-SRC and YES under serum-starved conditions (in the absence of EGFR phosphorylation Supp. Fig. 2). Binding of c-SRC and YES is reduced in the presence of FBS. Raw data blots shown in Supp. Fig. 3. (d) Quantification of immunoprecipitated SRC (left panel) and YES (right panel): N = 3, derived from densitometry measurement of western blotting in **(c)**.

The median number of interactors identified across the studied cell lines was 7 (range 0–14) for EGFR and 10 (range 0–14) for FGFR2, compared with 3 (range 0–14) for HER2. Given the relatively smaller number of interactors and LCIs identified for HER2, we sought to mitigate against any impact from the screening approach by undertaking an orthogonal approach using recombinantly expressed SH3 domains immobilised on a chip and monitoring binding to a fluorescently labelled HER2 (Fig. 4a, control data in Supp. Fig. 1). This identified additional interactions with FYN (which is a Src family kinase with high sequence homology with LYN which was previously characterised [21]) and PLCƳ1 but no other assessed SH3 domain-containing proteins.

By mass spectrometry, the most commonly identified SH3 domain-containing interactor was SPTAN1 followed by multiple motility and cytoskeletal modifying myosin proteins, including modifying myosin 9 (MYH9), myosin 14 (MYH14) and myosin 6 (MYO6) proteins, as well as the unconventional myosin 18A (MY18A) protein. There were no SH3 domain-containing interactors for EGFR or HER2 in squamous lung H520 cells or for FGFR2 in the control HEK293T cells.

Amongst these proteins, the most overrepresented processes (Fig. 4b) related to cellular homeostasis (“cellular process (GO:0009987)”, “biological regulation (GO:0065007)”, “multicellular organismal process (GO:0032501)”, “response to stimulus (GO:0050896)”), cellular invasion and cellular migration (“localisation (GO:0051179)”, “biological adhesion (GO:0065007)”, “developmental process (GO:0032502)”). The cellular component term ‘plasma membrane’ was associated with 34 (83 %) of the 41 PRM-SH3 interactors, representing statistical overrepresentation (false discovery rate, FDR 1.44 × 10^−10^).

The interactions reported here are fundamentally dependent on the relative concentrations of the RTKs and their binding partners, and their ability to localise within a given cell to compete for the receptor PRM. Thus, environmental conditions, both outside and within the cell, will dictate the complement of binding proteins observed. Our analysis of PRM-binding ligands does not fully represent the potential for promiscuity in binding of proteins because it can only represent the relative expression levels that exist in cell lysates under the conditions of the experiment. A change in concentration of a given SH3 domain-containing protein or a RTK by a few-fold might lead to the complement of affinity purified proteins being modified. This is exemplified by the absence of the previously reported binding of GRB2 and PLCƳ1 to FGFR2 in HEK293T in this study [19,20]. In earlier studies the PLCƳ1-FGFR2 interaction was observed in HEK293T cells when the adaptor protein GRB2 was knocked down and the low endogenous expression of FGFR2 was enhanced by stable transfection of the receptor [19].

Further to this, the approach adopted herein exposes the bait peptides to whole cell lysates and hence potentially removes the influence of intracellular compartmentalisation. As a result, some interactions we observe might be precluded through restricted access to cellular organelles. However, it is important to highlight that many RTKs are known to become internalised and translocate to a range of different cellular locations in response to various cellular ques. Hence, we cannot disregard all of the interactors that are not typically localised to the cytoplasm.

### 2.4 Validation of interaction between EGFR and c-SRC/YES

The SH3 domain-containing Src family proteins SRC and YES were both identified by AP-MS as EGFR interactors in HEK293T cells (Table 2). These proteins were selected to exemplify our interactome interactors because of extensive reported characterisation of Src family SH3 domain interactions [34], the requirement for reduced stringency for detection, and their potential importance in being able to initiate downstream signalling (including cancer signalling) on binding to an RTK. We immunoprecipitated EGFR after an 18-h period in which cells were cultured under serum-starved conditions without foetal bovine serum, FBS (i.e., in the absence of growth factor). These conditions are commonly reported to replicate basal, non-phosphorylated RTK conditions (e.g. [17]). The cells were then either exposed to FBS or persistently starved. The interactions of the SH3 domains from both SRC and YES with non-phosphorylated EGFR were confirmed in serum-starved cells (Fig. 4c). Both SRC and YES have SH3 and SH2 domains, however serum-starvation negates receptor phosphorylation and hence SH2 binding sites (control data Supp. Fig. 2). The addition of FBS reduces the binding of the Src family proteins to the receptor. The reason for this is not clear, however, it could reflect that SRC and YES are recruited by other receptors that are activated in the presence of low levels of stimulating ligands in FBS.

Further validation of a subset of interactions observed in Table 2 can be found in the literature where interactions between the RTKs studied herein and SH3 domain-containing proteins are reported (see Biogrid (https://thebiogrid.org)). In the absence of canonical interactions between pTyr and SH2 domains, these reported interactions can be inferred to be between the RTK-PRMs and SH3 domains in the respective proteins. For example, the following interactions are cited: EGFR with Myosin-9 [38]; ZO-1 [39]; Src substrate cortactin [40]; Sortin nexin-9 [40]; SH3 domain-containing protein 19 [14]; Brain-specific angiogenesis inhibitor1-associated protein 2 [41]; FGFR2 with Dystonin [14] and HER2 with Transport and Golgi organisation protein 1 homolog [14].

## 3 Discussion

Signalling derived from RTKs is implicated in the development and progression of multiple malignancies [2]. For the most part, the oncogenic action of RTKs has classically been regarded to result from kinase activity upregulation in response to mutation or a surfeit of growth factor. Despite this, there is evidence that overexpression of wild-type RTKs also closely correlates with outcomes in a number of malignancies [2]. Whilst there is some evidence that the increased local concentration of RTKs that results from their overexpression drives signalling, the heterologous mechanisms underlying this are not well delineated. There is, nevertheless, a growing body of evidence for the presence of a diverse and functionally important, but as yet poorly mapped, RTK interactome [14].

We have previously demonstrated that in conditions of relative RTK excess, a PRM sequence incorporated within the C-terminal tail of FGFR2 induces activation of intracellular effectors through SH3 domain-mediated interactions that occur in the absence of tyrosine kinase upregulation, and which associate with disease outcomes in a number of cancers [[18], [19], [20]]. We expand on this early work here by analysis of the interactome for a PRM from each of the RTKs; EGFR, FGFR2 and HER2, in cell lines derived from four cancers that together represent a diverse range of histological subtypes.

Overall, a greater number of PRM interactors were identified by affinity purification mass spectrometry for EGFR and FGFR2 than for HER2 (Fig. 2a). This reflects a relatively lower ability of the HER2 10.13039/100021602PRM to interact with intracellular proteins, which is supported by the small number of interactors bound by protein domain microarray (Fig. 4a). These data may suggest that some RTKs more freely dictate Tier 2 signalling than others. The C-terminus of HER2 includes two canonical SH3 domain binding PRMs and five other PRMs (Supp. Table 1) so our data presented here do not cover the entire range of possible Tier 2 interactions. Furthermore, the propensity of any given PRM interaction is likely to be dictated by differences in access to the RTK PRM that result from the conformation of the overall tail sequence within the cellular environment [42]. Underscoring this point, it should also be highlighted that, in this study we selected individual representative PRMs from the three RTKs, however each RTK does have other PRMs which could also provide binding sites (see Supp. Table 1). Therefore, further analysis of other PRMs might reveal a larger role for HER2 in Tier 2 signalling.

The interaction of a given domain with a PRM in cells is entirely dependent on its relative concentration with respect to other competing domains, and its cellular localisation. Interestingly, our data does appear to show a connection between the intracellular concentration of domain-containing proteins that dictates the likelihood of their PRM-mediated interaction. This is in keeping with our previous work [[17], [18], [19], [20]] and is coupled with evidence provided here that a majority of interacting proteins are found at the cell surface. This highlights a complex signalling dynamic in which both the specific RTK PRM and the local cellular environment in which it is found direct the signalling outcomes.

Amongst the interactors identified by mass spectrometry for EGFR, FGFR2 and HER2, contributors to metabolic, homeostatic and migratory processes were overrepresented. Whilst this was broadly reflected by the specific pathways overrepresented amongst the interactor peptides, additional immune processes were seen for at least the EGFR C-terminal PRM in U251 GBM cells. The propensity of these processes appearing might suggest that in non-pathological conditions Tier 2 signalling is responsible for ‘house-keeping’ and ‘response to environmental stress’ functions which can be fine-tuned and potentially reversed. This is in contrast to Tier 1 signals that tend to result in profound and irreversible cellular outcomes such as differentiation, proliferation and cell death [1].

Given that we have previously demonstrated a specific role for the SH3 domain in mediating interactions with RTK C-terminal PRMs [[17], [18], [19], [20]], we sought to additionally identify interactors with this domain for each of our studied RTKs. In doing so, we identified 41 SH3 domain-containing proteins of potential interest. Amongst these, processes relating to migration, invasion and homeostasis were overrepresented. More broadly, the overrepresentation of migratory and homeostatic terms amongst the identified interacting proteins may provide evidence for the ability of cancer to hijack an evolutionarily conserved mechanism. It would, for example, be beneficial for cells lacking in growth factor stimulation to upregulate survival pathways and potentially even migrate to an area of greater RTK ligand availability or escape unfavourable environmental conditions. This would occur via the Tier 2 mechanism demonstrated here but could potentially also be hijacked by a cancer cell through upregulation of RTK and/or downstream effector expression in response to a stressor that results in a relative excess of RTK tail PRM regions. Indeed, it is potentially possible that the change in expression profile of Tier 2 signal-initiating effectors could be stimulated by cellular response to therapeutic intervention, hence providing a mechanism for resistance.

In support of the presence of this mechanism, many of the specific interactors to the studied RTKs are already recognised to contribute to adverse cancer outcomes. We have, for instance, validated an interaction between the EGFR PRM and the SH3 domain-containing proteins SRC and YES. Dysregulation of the Src family kinases is well recognised in cancer and contributes to poorer outcomes [24]. This has typically been regarded to result from increased EGFR transactivation, but the work here suggests an additional mechanism through which the EGFR/Src family kinase proteins may contribute to deleterious cancer outcomes. Favouring signalling via this mechanism, lipid rafts have been shown to provide a platform for EGFR and c-SRC interaction in breast cancer cells [36].

Another interactor of potential interest is the EVH1 domain-containing switch associated protein 70 (SWAP70), which associated with EGFR in GBM U251 cells. This has been shown to mediate GBM migration and invasion by regulating CD44 expression [43].

This study provides evidence for the basis of Tier 2 interactions which, along with the limited number of these interactions that have now been comprehensively evaluated, require further validation. Furthermore, the weak and transient nature of SH3-PRM interactions is such that only a limited number of SH3 domain-containing proteins could be identified with high levels of confidence given the reliance of an AP-MS approach on stable and reasonably strong interactions. To this end, it would be appropriate to build on the work shown here with additional functional studies and by correlating the expression of relevant SH3 domain-containing proteins with survival in existing clinical datasets.

Our data, therefore, add considerably to contemporary developments in our understanding of the physical and functional interactome of human RTKs. Furthermore, we highlight that SH3 domains are likely to play a far more important and independent role in intracellular signalling than generally considered. In the absence of the requirement for an on/off functionality represented by tyrosine phosphorylation in SH2 domain-mediated signalling, signalling is dependent on concentration fluctuations of SH3 domain-containing proteins. This suggests roles in responding to environmental stress and metabolic and homeostatic regulation. However, under conditions of aberrant or prolonged response, oncogenic signalling can prevail. In the light of this, we might need to consider alternative therapeutic approaches to cancer and directed kinase inhibitor resistance.

The weak and transient nature of SH3-PRM interactions means that they are hard to identify using previously adopted protocols and stringency ‘cut-offs’ for proteomics studies. Therefore, this study is likely to underrepresent the breadth and number of interactions maintained by the RTK PRM. There are also clear but previously under-recognised differences in the ability of AP-MS scoring systems to identify these interactions, with a greater number of possible interactors identified in this study using Perseus rather than SAINTexpress. Clearly, this study is limited to PRMs from a subset of RTKs and five cell lines under one set of conditions. To truly reflect the possible interactions of the RTK-PRM transcriptome a more substantial screen would be required which would include multiple cell lines, representative physiological conditions and extensive validation of physiological and pathological relevance.

## 4 Methods

### 4.1 Materials and reagents

RIPA Lysis and Extraction Buffer (#89900) was obtained from Thermo Fisher Scientific or made in-house from the following: 1 % NP40 (GenTex, Irvine), 1 % Na-Deoxycholate (Sigma-Aldrich, St. Louis), 0.1 % SDS (Sigma-Aldrich, St. Louis), 0.15M NaCl (Honeywell, Seelze), 0.01M Na-phosphate (Sigma-Aldrich, St. Louis), 2 mM EDTA (Sigma-Aldrich, St. Louis), 50 mM NaF (Sigma-Aldrich, St. Louis) and 0.2 mM Na-orthovanadate (Sigma-Aldrich, St. Louis) at pH 7.2.

Bovine serum albumin (BSA; A9647) was purchased from Merck.

### 4.2 Mammalian cell culture

Human cell lines representing BrAC (SK-BR-3, ATCC HTB-30™) and LSCC (NCI–H520, ATCC HTB-182™), in addition to a control human embryonic kidney (HEK)-293T cell line, were obtained from the American Type Culture Collection (ATCC; Virginia, USA). The highly-transfectable HEK293T cell line was generated by stably transfecting HEK293T cells with FGFR2, as has previously been described [17]. This cell line has been extensively utilised to study the impact of the upregulation of FGFR2 and other endogenously expressed RTKs [44]. Human cell lines representing OAC (OE19, JROECL19) and GBM (U251 MG, #89081403) were obtained from the European Collection of Cell Cultures (ECACC; Salisbury, UK). All cells were assessed for mycoplasma contamination at monthly intervals using the LookOut Mycoplasma Detection kit (MP0035, Thermo Fisher Scientific, Loughborough, UK).

Each cell line was maintained as a sub-confluent culture at 37 °C in a humidified atmosphere with 5 % carbon dioxide (CO_2_) in air. OE19 and H520 cells were maintained in Roswell Park Memorial Institute (RPMI)-1640 growth medium (R6504, Sigma Aldrich, St Louis., USA) supplemented with 10 % (v/v) foetal bovine serum (FBS; Sigma Aldrich) and 2 mM l-glutamine. SK-BR-3, U251-MG and HEK293T cells were cultured in Dulbecco's Modified Eagle Medium (DMEM) supplemented with 10 % (v/v) FBS, 50 μg/ml gentamicin and 7 μg/ml puromycin (Sigma Aldrich). When not passaged, 50 % media exchanges were undertaken at three-day intervals.

### 4.3 Cell lysate and sample preparation

Prior to lysis and streptavidin pulldown using wild-type or scrambled RTK C-terminal tail sequences, cells were grown to around 90 % confluence in a 100 mm tissue culture dish. In order to maintain cellular viability, each cell line was maintained in RPMI/DMEM supplemented with 10 % FBS, as outlined above. Though FBS does contain growth factors and may therefore facilitate classic ligand inducible RTK activation, RTK loop phosphorylation in the presence of FBS is nevertheless known to be low and conditions of serum (i.e., FBS) starvation are associated with cellular stress (e.g., FGFR2 [17]). Furthermore, none of the wild-type or scrambled bait peptides contained ligand-inducible tyrosine residues, thereby favouring PRM-mediated interactions even in the presence of supplemented growth factor.

Prior to lysis, cells were washed three times in ice-cold phosphate buffered saline (PBS) and harvested in RIPA Lysis & Extraction buffer (see above). Cells were further disrupted and homogenised via hydrodynamic shearing using a 0.8 mm needle followed by 1 h of continuous rotation at 4 °C. Cell debris and DNA were subsequently removed through aspiration of the supernatant following by centrifugation at 2000*g* for 15 min at 4 °C. Sixteen cell pellets were independently prepared for each cell line. Protein concentration was determined using a colorimetric Pierce™ BSA Protein Assay Kit (#23225, Thermo Fisher Scientific). Lysates were subsequently stored at −80 °C prior to experimentation.

### 4.4 Biotinylated bait peptides

The presence of large affinity tags, particularly when fused to small peptide sequences, can impact on the structure and therefore function of the proteins to which they are bound [45]. In contrast, conjugation of biotin is unlikely to impact on function given its small size. Synthetic peptides representing the PRM-containing cytoplasmic tail sequence of EGFR (VQNPVFHNQPLNPAPSRDPH – residues 1105–1124), HER2 (DVRPQPPSPREGPLPAAR – residues 1144–1161) and FGFR2 (EPSLPQFPHINGSVKT – residues 806–821) were therefore commercially prepared and modified through the covalent N-terminal addition of a biotin tag (GenScript Biotech, The Netherlands). Scrambled control sequences that did not contain a PRM were similarly prepared for EGFR (VQNLVFHNQLLNLALSRDLH), HER2 (DVRLQLLSLREGLLLAAR) and FGFR2 (ELSLLQFLHINGSVKT). Tyrosine residues (Y1110: EGFR and Y812: FGFR2) in RTK sequences were mutated to phenyl alanine (F1110 and Y812 respectively) to remove any opportunity for phosphorylation. In all cases, peptide sequences were separated from the biotin tag by two inert, highly hydrophilic polyethylene glycol (PEG) spacers in order to increase solubility. PEG spacers are known to have minimal impact on the conformational properties of small neutral peptides [46].

### 4.5 Pull down and affinity enrichment

Each biotinylated tail sequence and matched scrambled control was assayed in biological triplicate in each cell line. Protein extracts were pre-cleared through the addition of 1 mg total protein lysate to 10 μl streptavidin agarose (Pierce, 88817) beads for a period of 1 h. Streptavidin beads for protein elution were pre-incubated with 50 μg peptide in 100 μl RIPA buffer at 4 °C for 1 h. The streptavidin beads were removed from the pre-cleared protein solution by centrifugation and the pre-incubated streptavidin beads subsequently added. Samples were incubated overnight at 4 °C with constant agitation. Following this, the streptavidin beads were pelleted by centrifugation and the supernatant discarded. The now protein-bound beads were subsequently washed twice in RIPA buffer and the beads stored at −80 °C.

Protein elution proceeded though the incubation of streptavidin beads with 30 μl 20 mM dithiothreitol in a 5 % sodium dodecyl sulphate (SDS), 50 mM Tris-HCl (pH 7.6) buffer at 90 °C for 10 min. Proteins were alkylated through the subsequent incubation for 30 min in the dark with iodoacetamide to a final concentration of 150 mM. Samples were then prepared for mass spectrometry by protein tryptic digest using the Suspension Trapping (Strap) method for bottom-up proteomics analysis, as has previously been described [45].

### 4.6 Liquid chromatography mass spectrometry

Processed peptides were analysed by nanoflow liquid chromatography mass spectrometry (LC-MS) using an EASY-nLC 1000 Liquid Chromatograph (Thermo Fisher Scientific) connected to a custom-made 30-cm capillary emitter column (75 μm inner diameter, 3 μm Reprosil-Pur 120C18 media). Mass spectrometry analysis was performed on a linear quadrupole ion trap - orbitrap (LTQ-Orbitrap) Velos mass spectrometer (Thermo). Total acquisition time was set to 100 min, with a gradient of 3–22 % acetonitrile in 0.1 % formic acid. For the survey scan, the resolving power was set at 60,000 with a scan range of 305–1350 amu. MS/MS data were obtained by fragmenting up to the twenty most intense ions in the linear ion trap. Data were searched against the Uniprot human protein sequence database with MaxQuant software package (www.maxquant.org) [47]. The maximum protein and peptide false discovery rates were set to 0.01.

### 4.7 Probabilistic modelling for scoring AP-MS data

Significance Analysis of INTeractome express (SAINTexpress) software was used to estimate the probability that each postulated bait-prey protein-protein interaction from the AP-MS data was true [48]. This probabilistic model is not vulnerable to quantitative variation of prey proteins across studied purifications and additionally accounts for negative control purifications such as those used here; thereby robustly removing background noise whilst accommodating for the impact of random sampling. A final interaction score (AvgP) for each bait-prey protein-protein pair is then calculated by averaging the probabilities for individual replicates.

Here, AP-MS data for each bait peptide were examined separately using SAINTexpress version 3.1.0 (http://saint-apms.sourceforge.net). Final AvgP results of 0.5 or greater were retained for further analysis. Common contaminants were removed using the peer-annotated Contaminant Repository for Affinity Purification-Mass Spectrometry data (CRAPome), version 2.0 (http://crapome.org) [49]. This uses mass spectrometry data from 716 experiments to filter possible contaminants. Only proteins with a CRAPome frequency of less than 358/716 (50 %) were retained. These interactors were subsequently explored using functional annotation. A separate group of low confidence interactors (LCIs) with an AvgP score of greater than 0 but less than 0.5 and a CRAPome frequency of less than 50 % were also identified for analyses relating to the interaction of SH3 domain-containing proteins with PRMs.

A second approach to the identification of SH3-containing binding partners was undertaken using Perseus (2.0.3.0) [37]. This computational platform uses peptide intensity-based quantification to identify proteins that are enriched in the presence of specific bait peptides, with a permutation-based false discovery rate applied for each sample-control pair. Interactors were identified here using a two-sample *t*-test with an FDR cut-off of 0.05.

### 4.8 Functional annotation

Protein domains were manually annotated using HumanMine v12 [50]. Conserved Pfam protein domains present in each HCI were identified using the Ensembl BioMart data mining tool (https://uswest.ensembl.org/info/data/biomart/index.html) [51]. Identified Pfam domains were grouped into Clans [52]. Searches were restricted to superfamilies. Over-represented Gene Ontology (GO) terms were identified from interactors using Protein Analysis Through Evolutionary Relationships (PANTHER) version 16.0 [53]. Functionally enriched GO Biological Process (BP), Molecular Function (MF) and Cellular Component (CC) terms were identified using a reference *Homo Sapiens* gene set.

### 4.9 Protein domain microarray

A protein-domain microarray was used in order to identify potential SH3 domain-containing interactors with the HER2 receptor. The use of this system to identify novel protein-protein interactions has been described previously [54]. Briefly, the experimental workflow includes purification of glutathione S-transferase (GST) fusion SH3 domain-containing proteins, the arraying of these proteins on a microarray and their probing using PRM-containing RTK tail sequences with interactions determined using fluorescent probes.

### 4.10 Purification of GST fusion proteins

Overexpression of glutathione S-transferase (GST) fusion proteins was induced in DH5α *Escherichia coli* cells (Life Technologies, MD, USA) using 0.4 mM isopropyl β-d-thiogalactopyranoside and cells subsequently broken by sonication. GST fusion proteins were extracted from the resultant lysates by centrifugation at 12000*g* for 10 min followed by binding to glutathione-Sepharose 4B beads (Amersham Pharmacia Biotech, NJ, USA). Purified proteins were eluted using 30 mM glutathione, 50 mM Tris/HCl, pH 7.5 and 120 mM NaCl then stored at −70 °C.

### 4.11 Protein microarray peptides

A protein microarray incorporating 30 SH3 domain-containing protein sequences was generated as outlined previously [55]. Approximately 250 ng of each protein stock was arrayed on to one of 25 specific spots on a nitrocellulose pre-coated glass FAST™ slide (Schleicher & Schuell, NH, USA). Spots were spaced at a 700 μm distance from one another, and each protein was spotted in duplicate then allowed to air dry. A control GST-alone spot was placed in the centre of this grid.

### 4.12 Protein microarray probes

Biotinylated peptides representing the PRM-containing HER2 tail sequence (GGGGAAPQPHPPPAFSPAFDNL) and the non-PRM IGF1R (GGGGRKNERALPLPQSST) tail sequence were synthesised by Genscript (NJ, USA). Each was then bound to 5 μl Cy3-streptavidin (Fluorolink™; Amersham Pharmacia Biotech) in 500 μl PBS containing 0.1 % Tween 20; PBST) and then incubated with 20 μl biotin-agarose beads (Sigma, MO, USA).

### 4.13 Probe-peptide interaction

Arrayed slides were blocked in PBST containing 3 % (w/v) powdered milk within an Atlas Glass Hybridisation Chamber (Clontech, CA, USA) then hybridised to 400 μl fluorophore-tagged peptide for 1 h. Three 10 min washes with PBST were subsequently used to remove unbound peptide and the slide dried via centrifugation. Following this, a 550 nm long pass filter was used for the detection of the Cy3-labelled probes via a GeneTAC™ LSIV scanner (Genomic Solutions). Fluorescence of two dots representing the same protein is regarded as a positive indication of peptide-probe interaction.

### 4.14 Immunoprecipitation and western blotting

HEK 293T cells were grown to 80 % confluency and then cultured in media not supplemented with FBS for 18 h. Following this period, cells were cultured for 45 min with and without FBS supplementation. Cells were lysed in lysis buffer (50 mM HEPES, 50 mM NaCl 1 mM EGTA, 10 % (w/v) glycerol, 1 mM sodium orthovanadate, 10 mM sodium fluoride, 0.1 % NP-40, supplemented with protease inhibitors) and cleared of cell debris via centrifugation.

Following quantification of protein concentration, 1 mg of cell lysate was incubated at room temperature with 10 μg *anti*-EGFR (SCBT; sc-120-AC) or 10 μg anti-mouse IgG (SCBT; sc-2343) for 2 h at room temperature, with gentle rotation. Immunoprecipitants were subsequently washed three times with 1 ml lysis buffer. Samples were then analysed by western blotting. Antibodies used in western blotting were EGFR (CST; cat no. 4267), Src (CST; cat no. 2123), Yes (CST; cat no. 3201).

### 4.15 Correlation of binding patterns with expression profiles

In order to determine whether the interactors identified via AP-MS correlated with the specific expression profile of these interactors in each cell line, we extracted mRNA expression data from the Cancer Cell Line Encyclopedia (CCLE) [33,56]. Specifically, gene expression transcript per million (TPM) values of protein coding genes for all DepMap cell lines, reported using a pseudo-count of log2(TPM+1), were downloaded from DepMap Public 23Q2 primary files (file: ‘OmicsExpressionProteinCodingGenesTPMLogp1’). A detailed description of the pipelines used to generate these expression data can be found at https://github.com/broadinstitute/ccle_processing#rnaseq. Data relating to the studied cell lines were extracted using the following DepMapIDs: ACH-000017 (SK-BR-3), ACH-000679 (OE19), ACH-000049 (HEK293T), ACH-000232 (U251) and ACH-000395 (NCIH520). We then searched within these datasets for genes corresponding to all SH3-domain proteins identified to be a possible interactor in at least one cell line (these are summarised in Table 2). The pseudo-count of these genes is shown for each cell line and correlated against the presence or absence of an interaction with any of the studied RTKs in that cell line (i.e. counts are provided either for ‘no interaction’, in which the specific interactor from the studied cohort did not bind to any of the RTKs studied in that cell line or ‘interaction’, in which the specific interactor bound to at least one of the RTKs in that cell line.

## Illustrations

Illustrations were created using Servier Medical Art, provided by Servier, licensed under a Creative Attribution 3.0 unreported license. Unless otherwise stated, graphs have been generated using GraphPad Prism 8.1.2. (GraphPad Software, CA, USA).

## Funding information

This work was supported by a 10.13039/501100000289Cancer Research UK (10.13039/501100000289CRUK) Program Grant awarded to JEL (C57233/A22356). CMJ was supported for the duration of this work by a 10.13039/100010269Wellcome Trust N4 Clinical Research Training Fellowship jointly held by the Universities of Leeds, Manchester, Newcastle and Sheffield (203914/Z/16/Z) and by a Clinical Lectureship part funded by 10.13039/501100000289CRUK RadNet Cambridge (C17918/A28870). Probing of arrayed SH3 domains was made possible through the 10.13039/100007313University of Texas MD Anderson Cancer Center Protein 10.13039/100007174Array & Analysis Core (10.13039/100029971PAAC), supported by 10.13039/100004917CPRIT Grant RP180804 (10.13039/100004768MTB).

## Ethics declaration

Review and/or approval by an ethics committee was not needed for this study because no experiments or data acquisition involved patients or patient samples.

## Data availability

Mass spectrometry proteomics data have been deposited to the ProteomeXchange Consortium via the Proteomics IDEntifications Database (PRIDE) partner data repository with the dataset identifier PXD041383.

## CRediT authorship contribution statement

**Christopher M. Jones:** Writing – review & editing, Writing – original draft, Validation, Investigation, Formal analysis, Data curation, Conceptualization. **Arndt Rohwedder:** Investigation, Formal analysis. **Kin Man Suen:** Investigation, Formal analysis. **Safoura Zahed Mohajerani:** Investigation, Formal analysis. **Antonio N. Calabrese:** Writing – review & editing, Methodology, Conceptualization. **Sabine Knipp:** Formal analysis. **Mark T. Bedford:** Methodology, Investigation, Formal analysis, Conceptualization. **John E. Ladbury:** Writing – review & editing, Writing – original draft, Supervision, Resources, Project administration, Investigation, Funding acquisition, Conceptualization.

## Declaration of competing interest

The authors declare that they have no known competing financial interests or personal relationships that could have appeared to influence the work reported in this paper.
