# Supplementary figures and images for: Affinity purification mass spectrometry characterisation of the interactome of receptor tyrosine kinase proline-rich motifs in cancer
Source: Heliyon. 2024 Jul 31;10(15):e35480. doi: 10.1016/j.heliyon.2024.e35480 (PMC11334840; doi:10.1016/j.heliyon.2024.e35480)

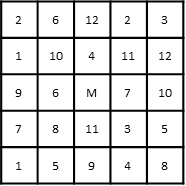

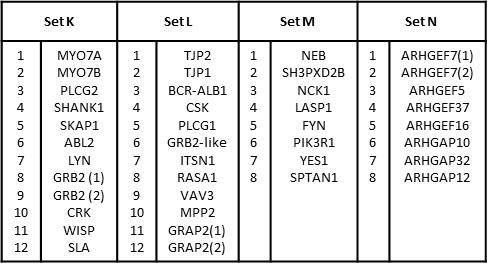

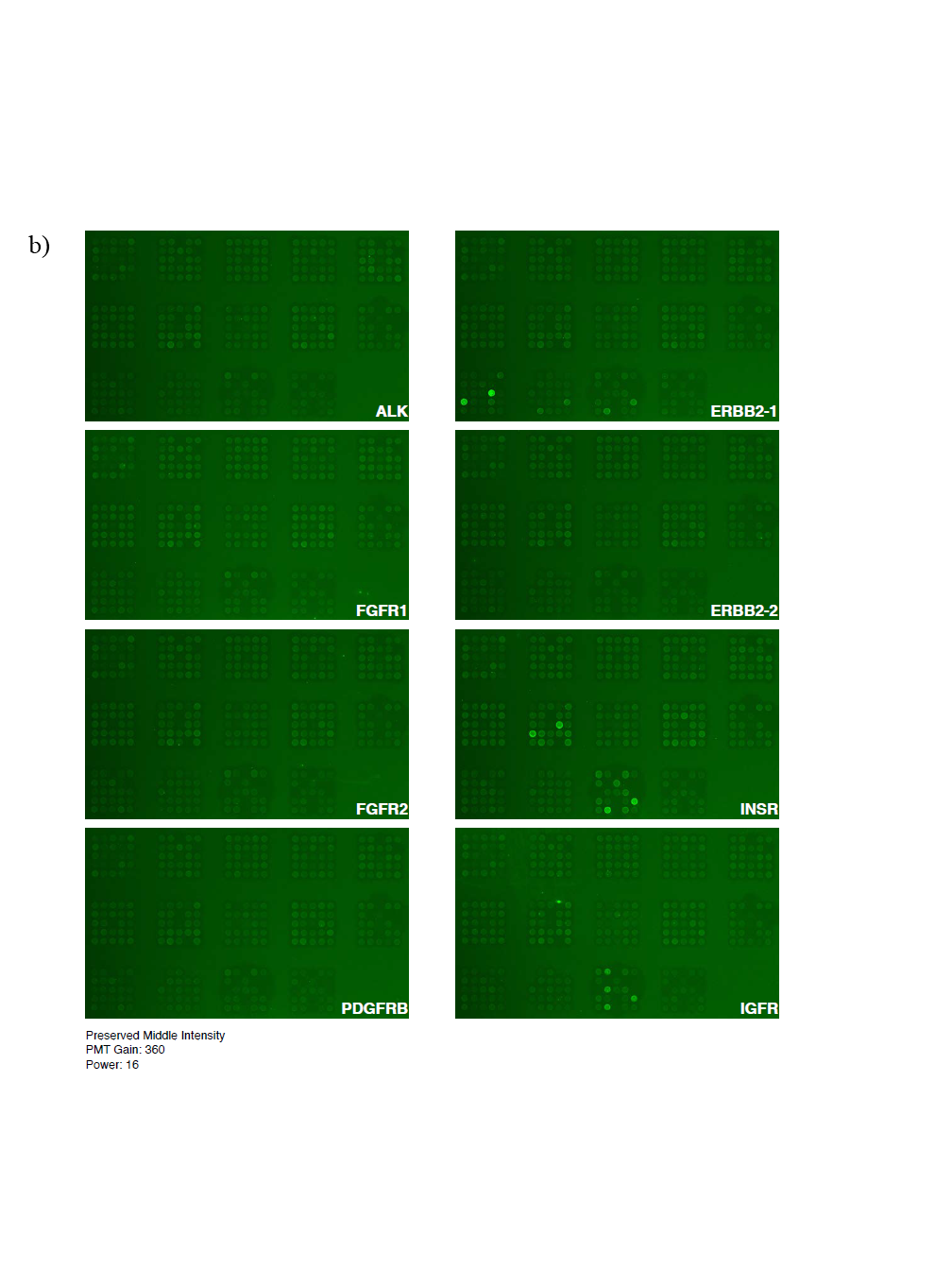

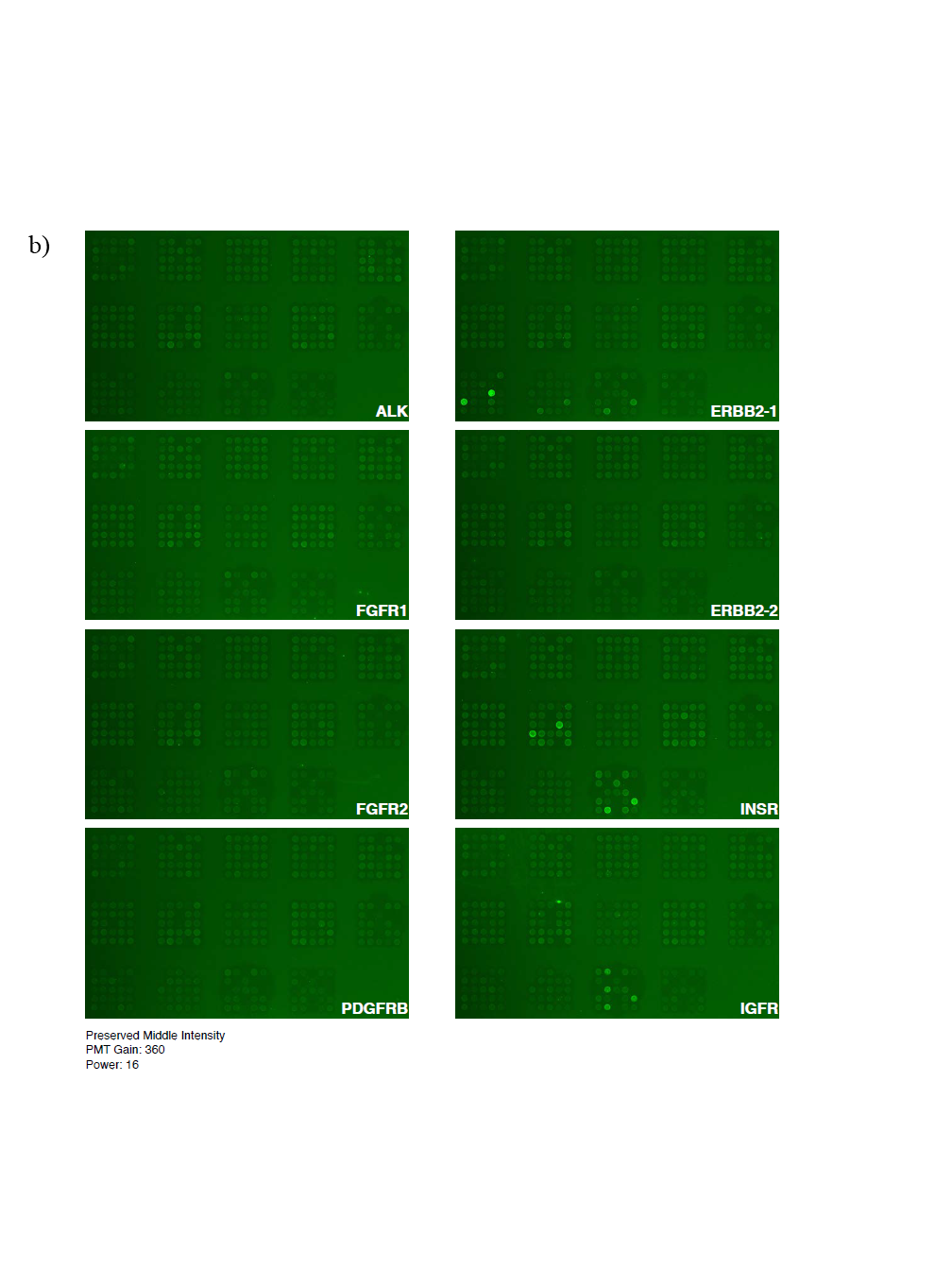


**Supplementary Figure 1**

Set K

Set L

Set M

Set N

**Supplementary Figure 2**


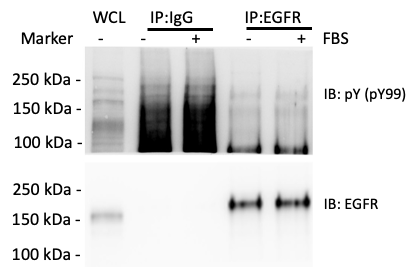


**Supplementary Figure 3**

a)


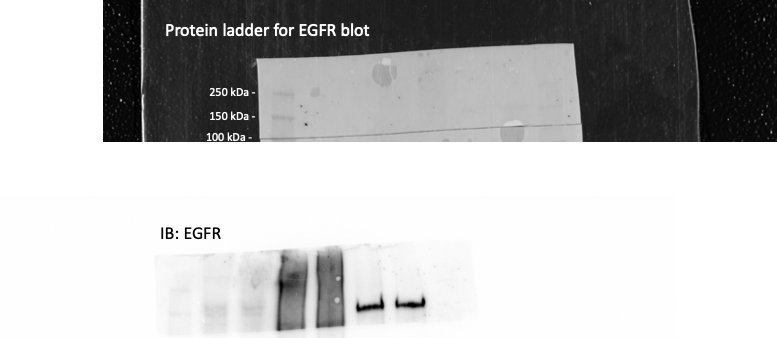


b)


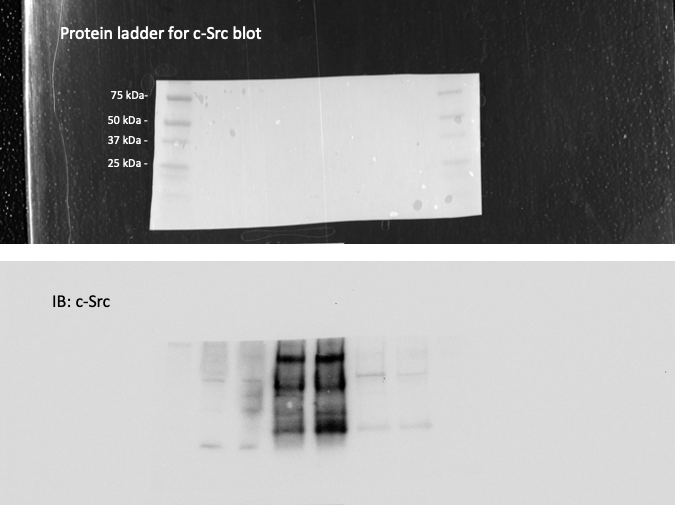


c)


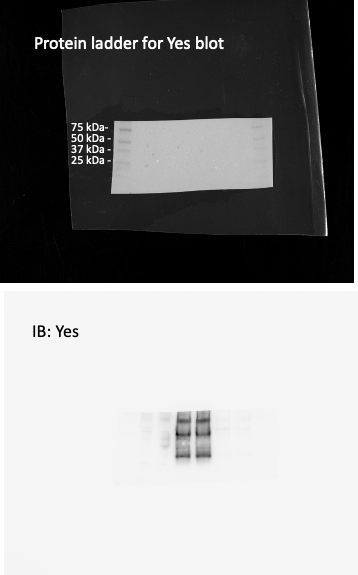


d)
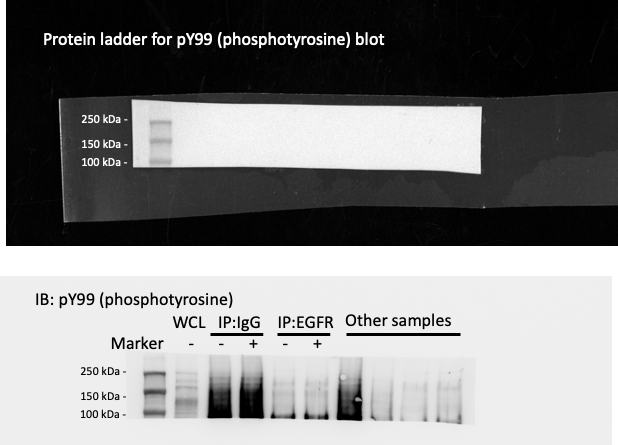

Supplement: Multimedia component 1 [file mmc1.docx]
